# Percentile Reference Values for the Neck Circumference of Mexican Children

**DOI:** 10.3390/children8050407

**Published:** 2021-05-18

**Authors:** Evelyn Valencia-Sosa, Clío Chávez-Palencia, Juan R. Vallarta-Robledo, Enrique Romero-Velarde, Alfredo Larrosa-Haro, Edgar Manuel Vásquez-Garibay, César Octavio Ramos-García

**Affiliations:** 1 Instituto Tecnológico de Estudios Superiores de Occidente, Anillo Periférico Sur Manuel Gómez Morín 8585, Santa María Tequepexpan, Tlaquepaque, Jalisco 45604, Mexico; envs_621@hotmail.com (E.V.-S.); cybervallarta@hotmail.com (J.R.V.-R.); 2 Departamento de Salud Pública, Centro Universitario de Ciencias de la Salud (CUCS), Universidad de Guadalajara, 950 Sierra Mojada St. Guadalajara, Jalisco 44340, Mexico; 3 División de Ciencias de la Salud, Centro Universitario de Tonalá, Universidad de Guadalajara, Av. Nuevo Periférico 555 Ejido San José Tatepozco, Tonalá, Jalisco 45425, Mexico; cesar.ramos@ciansc.com; 4 Instituto de Nutrición Humana, Universidad de Guadalajara, Salvador Quevedo y Zubieta 750, Guadalajara, Jalisco 44340, Mexico; enrique.rvelarde@academicos.udg.mx (E.R.-V.); alfredo.larrosa@academicos.udg.mx (A.L.-H.); edgar.vgaribay@academicos.udg.mx (E.M.V.-G.)

**Keywords:** childhood, neck circumference, percentile, anthropometry

## Abstract

Neck circumference was studied for the first time in a pediatric population in 2010. Since then, various countries have proposed cutoff values to identify overweight, obesity, and metabolic syndrome. However, no reference values have been established for the Mexican child population. The aim of this study is to provide percentile reference values for the neck circumference of Mexican schoolchildren. Only normal-weight schoolchildren aged 6–11 years were included. Percentiles and growth charts were constructed based on the “Generalized Additive Model for Location, Scale and Shape” (GAMLSS). A total of 1059 schoolchildren (52.9% female) was evaluated. Weight, height, and BMI values were higher for males; however, this difference was not statistically significant. The 50th percentile for females was 24.6 cm at six years old and 28.25 cm at 11 years old, and for males, it was 25.75 cm and 28.76 cm, respectively. Both males and females displayed a pronounced increase in neck circumference between 10 and 11 years of age. The greatest variability was found in the 11-year-old group, with an increase of 5.5 cm for males and 5.4 cm for females. This study presents the first reference values for neck circumference for a Mexican child population.

## 1. Introduction

The prevalence of childhood obesity has increased worldwide in recent decades. According to the National Health and Nutrition Survey in Mexico (ENSANUT, 2018), three out of ten children are overweight or obese, whereas the prevalence in adolescents surpasses 38% [1]. These numbers place Mexico among the countries with the highest overweight/obesity prevalence in the pediatric population. Furthermore, it has been reported that prominent fat depots in the upper body increase the risk for metabolic disturbances to a greater extent than general adiposity [2]. Therefore, it is important to develop practical and noninvasive indicators to assess body fat distribution.

Body mass index (BMI) is the most practical and utilized index for assessing normal weight ranges; however, it has been proven to be unsuitable for determining fat mass volume and location [3,4]. On the other hand, waist circumference (WC) and waist-to-height ratio have been proposed as reliable tools to identify individuals at metabolic risk, as both reflect central adiposity [5]. Moreover, the fact that a few technical issues may arise when measuring WC has led to the study of novel indicators.

Neck circumference (NC) has been proposed as a simple, minimally invasive, and inexpensive indicator to identify upper-body adiposity. Research on NC began in 2010 and ever since, it has been shown a wide association with central adiposity [6] along the onset of metabolic alterations [7]. Several cutoff points have been proposed to identify overweight and obesity [8,9,10], non-alcoholic fatty liver disease (NAFLD) [11], hypertension [12] and metabolic syndrome [13]. Nonetheless, the usefulness of these values is limited to the screened population. In Mexico, we have previously demonstrated that NC shows a high correlation with WC, which indicates that it might be utilized for the identification of elevated central adiposity [14]. However, no reference values have been established for the Mexican child population. In this regard, the creation of new reference tables with percentile values distribution for neck circumference might be useful to determine how distant they are from the mean as for sex and age, which could facilitate the identification of individuals at risk in clinical practice. Thus, the aim of this study is to provide percentile reference values of neck circumference of Mexican schoolchildren.

## 2. Materials and Methods

### 2.1. Participants

The sample for this study was obtained as part of a broader project entitled “Active intervention to improve feeding habits and physical activity in school children” conducted in six different schools located in Acatlán de Juárez and Villa Corona, Jalisco, Mexico. The sample size of the original project was calculated on data from a similar study by Li et al. [15] obtaining a total sample size of 288 children among the 6 schools (3 control and 3 intervention, selected by convenience sampling), with an extra 30% due to possible dropouts. A type I error of 0.05 and power of 80% were considered. Nevertheless, anthropometric measurements were taken in all children from the six schools at baseline and final stages. Remarkably, the data for this study were obtained from the baseline stage. Invitation to participate was granted to 2070 students; however, the assessments were performed in 1802 children aged 6–11 years (the elementary education in Mexico comprises these ages), Nevertheless, only 1059 were included because data from normal-weight children are required to create reference values.

The inclusion criteria were as follows: children attending six elementary schools in Acatlán and Villa Corona Jalisco, Mexico from November 2015 to January 2016. Exclusion criteria consisted of being under any type of nutritional or medical treatment and/or having a chronic disease.

This study was conducted according to the guidelines of the Declaration of Helsinki. All procedures involving human subjects/patients were approved by the Comité de Ética en Investigación del Centro Universitario de Tonalá (003–2016). Verbal informed consent was obtained and formally recorded from all the subjects and their tutors. Written informed consent was obtained from all the school directors.

### 2.2. Measurements

All anthropometrical measurements were carried out by two trained researchers according to the Habitch method [16]. Height was measured using a portable stadiometer with a precision of 0.1 cm (SECA, Hamburg, Germany) with the subject shoeless and the child’s head held in the Frankfurt horizontal plane. Body weight was measured using a calibrated electronic weighing scale (SECA, Hamburg, Germany) with a precision of 0.05 kg with children shoeless and without heavy extra clothing such as sweaters and jackets. WC and NC were measured to the nearest 0.1 cm using a metallic tape (606PMMX, Apex Tool Group, Lufkin, Queretaro, Mexico). WC was measured at the midway point between the lowest rib and the top of the iliac crest with the subject standing and at the end of a regular expiration. NC was measured at the midpoint of the neck at the level of the thyroid cartilage and perpendicular to the neck axis with the participant’s body held erect, eyes facing forward, and breathing normally. The triceps and subscapular skinfold thickness [17] were used to estimate the body fat percentage (BF%) according to Slaughter’s equation [18]. BMI was obtained by dividing the weight in kilograms by the height in square meters.

All measurements were taken twice. However, when the height, weight, and circumference differed by 1% or more, or by 5% in the case of skinfold thickness, a third measurement was performed. The mean of these values was used for the analyses.

### 2.3. Operational Definitions of Terms

Underweight was indicated by a BMI for age < −2 SD, normal weight was a BMI between −2 and +1 SD, overweight was a BMI between +1 and +2 SD, and obesity was indicated by a BMI > +2 SD [19]. Only normal weight participants were included to assure that the reference curves represented “*a standard healthy population*” in accordance with the World Health Organization (WHO).

### 2.4. Statistical Analysis

To determine the distribution of the quantitative variables, the Kolmogorov–Smirnov normality test was used. The significance level was established at *p* < 0.05 for all hypothesis tests. Numerical variables are reported as the mean and standard deviation (SD). Comparisons were conducted between groups using Student’s *t*-test for independent samples. Pearson correlation coefficients were calculated to explore the associations between NC and anthropometric variables. The following classification was used to categorized r values: Low or weak correlations (<0.35), modest or moderate correlations (0.36 to 0.67), and strong or high correlations (0.68–1.0). However, r coefficients > 0.90 were considered as “very high correlations” [20]. A *p* < 0.05 was considered statistically significant.

Percentiles and growth charts for neck circumference were constructed based on the “*Generalized Additive Model for Location, Scale and Shape*” (GAMLSS) [21], which is an extension of the lambda-mu-sigma method (LMS) by Cole and Green (1992). This method allows the construction of smooth curves at different percentile intervals based on age when the distribution is not normal. LMS transforms the age value at a specific exponential; thus, it prevents the tendency for distortion due to the classic rapid growth that occurs at early age stages [22]. Lambda (L) represents the skewness, mu (M) reflects the median, and sigma (S) is equal to the coefficient of variation. In contrast to the LMS method, which is based only on skewness, the GAMLSS method applies two additional submethodologies: Box-Cox power transform (LMSP) and Box-Cox t (LMST). These submethodologies are also adjusted by kurtosis [23,24].

For this study, all three methods (LMS, LMSP, and LMST) were applied for each sex and age transformation. The most suitable model was selected based on the lowest value for the Akaike information criterion (AIC). To validate this model, Q–Q and worm plots were created, and the Filliben plot correlation coefficient was calculated. Then, all the values were verified to confirm that the mean, standard deviation, skewness, and kurtosis were close to 0, 1, 0, and 3, respectively [21].

After the model selection, percentile values were calculated: 3rd, 5th, 10th, 25th, 50th, 75th, 85th, 95th, and 97th by sex and age using the following formula:

X = M(1 + LSz) ^(1/L)

where,

X = percentile value

Z = z score

M = mu

S = sigma

L = lambda

Statistical analyses were performed using R (3.4.4, R Foundation for Stati, Vienna, Austria) and RStudio (1.2.1335, PBC, Boston, MA, USA) software. The GAMLSS package was used for the construction of growth charts [25].

## 3. Results

For this study, a total of 1059 schoolchildren aged 6 to 11 years were included (52.9% female). Weight, height, and BMI values were higher for males; however, this difference was not statistically significant. Similarly, males showed greater neck and waist circumferences than females (*p* < 0.05). On the other hand, the body fat percentage was higher for females (2.59% higher than males; *p* < 0.001).

Table 1 shows the anthropometric measurements of the studied population by sex and age. It also shows that NC and WC displayed higher values in males, except for the 11-year-old group, in which girls showed higher WC values compared to boys. 

The correlations between NC and all the anthropometric variables were statistically significant, regardless of sex and age. For WC and BMI, the correlations ranged from r = 0.5 to r = 0.8, whereas for BF% and skinfold thickness, the correlations had lower values, ranging from r = 0.2 to r = 0.7 (Table 2).

Regarding the percentile distribution for neck circumference, both males and females displayed a pronounced increase between 10 and 11 years of age. The 6-to-7-year-old group had the lowest increase. The greatest variability (97th percentile minus the 3rd percentile) was found for the 11-year-old group, with an increase of 5.45 cm for males and 5.39 cm for females.

The least variability was observed for the six-year-old children, with increments of 4.18 and 4.03 cm for the males and females, respectively. At the 50th percentile, the yearly increment of neck circumference ranged from 0.5 to 1.0 cm (Table 3).

**Figure 1:**
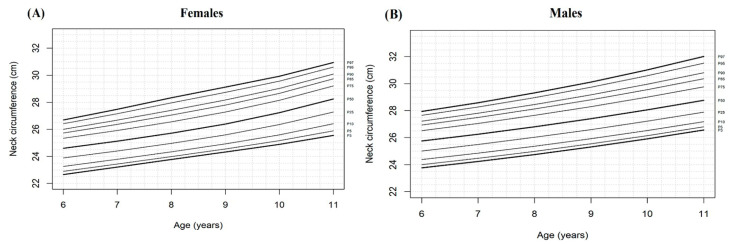
Percentiles of neck circumference of females (**A**) and males (**B**) aged 6–11 years in Acatlán de Juarez and Villa Corona Jalisco, México.

Growth charts for neck circumference showed a linear and constant tendency for both sexes starting with the 10-year-old group. Similarly, neck circumference was greater for males than for females (Figure 1).

## 4. Discussion

This study provides reference percentile values for neck circumference from a Mexican schoolchildren cohort. Remarkably, as of the submission of this report, there was no other similar study for the Mexican population. Given their correlation with upper body adiposity, our results may be applied as references in future research and in clinical practice to identify individuals at risk for overweight and obesity [14,26,27].

The correlation values we presented here were statistically significant but lower than those we previously reported (where overweight and obese children were included) [14]. This reinforces the hypothesis that the correlation values tend to be higher for these children. In general, BMI and WC were the variables with the highest correlation values with NC, which was consistent with other studies that included children, regardless of body weight [26,28,29,30].

Neck circumference was greater for the male than for the female participants, and it showed an age-dependent increase. The age group with the smallest increment was for children between 6 and 7 years old, whereas the most pronounced increment was for children between 10 and 11 years old for both sexes. This increment might be related to the onset of puberty, which normally begins at 10–11 years and 13–14 years for girls and boys, respectively. Most importantly, female participants had the greatest yearly increment, regardless of age, for neck circumference, waist circumference, and body fat percentage. This finding represents relevant evidence of the association between neck circumference and adiposity indicators in this gender.

The percentile values presented herein were similar to those reported by Katz [31] for Canadian children. These results can be explained by the fact that both samples included children of normal weight. Although the Canadian study presented data from 6-17-year-old participants, the values appear to be consistent with those in our study of Mexican children up to 11 years of age. The 50th percentile values for girls were slightly lower in our study, with differences ranging from 0.0 to 0.3 cm. Nevertheless, the values for boys were higher than those in Katz’s report, differing from 0.0 to 0.5. This was not the case for the report of European children by Nagy [32], who included normal-weight subjects, with NC values for both males and females 6–10 years of age displaying greater differences, ranging from 0.2 to 0.9 cm, than in our study.

Our data were also similar to those reported by Mazicioglu [26] in Turkey. The values of female subjects at the 50th percentile varied between 24.9 and 28.5 cm, close to the data reported herein (24.6 to 28.3 cm). Male participants showed a similar pattern, ranging from 25.6 to 28.8 cm versus 25.8 to 28.8 cm for the Turkish data and Mexican data, respectively. Notably, the values represent Turkish children between the 3rd and 97th percentiles for weight. Similarly, the percentiles reported by Hosseini for Iranian children showed values at the 50th percentile ranging from 26.3 to 28.3 cm for males and 25.4 to 28.2 cm for females [33]. Nonetheless, these percentiles were based on children who were 7 years of age and older, regardless of weight.

**Figure 2:**
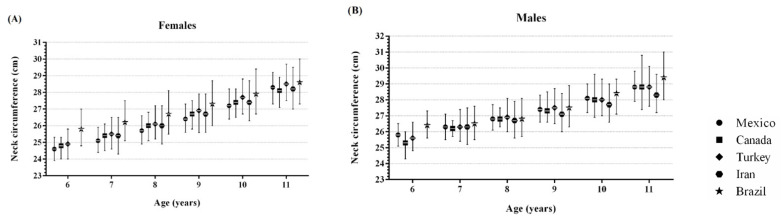
Comparison of 25th, 50th and 75th percentiles values for neck circumference (NC) among females (**A**) and males (**B**) from different countries. Note: Mexico and Canada [31] included normal weight children. Turkey [26] removed the extreme values by determining the 3rd–97th percentiles for weight. Iran [33] included the entire sample. Brazil [34] included children with NC values within ±3 SD.

On the other hand, Coutinho’s report [34] on Brazilian children showed higher values at the 50th percentile compared to those in our study. This difference was more notable for girls 6–8 years of age (0.3–1.2 cm). For boys, the difference was less (0.1–0.6 cm). However, these percentiles were calculated based on children with NC values within ±3 SD. A graphical comparison between the 25th, 50th and 75th percentiles for NC data from the aforementioned countries is shown in Figure 2.

According to the WHO, to create reference growth charts, it is necessary to include normal weight subjects only to represent the ideal increment. It is evident that the data we present showed lower values compared to most of the studies we cited, except for those studies that included only normal-weight children.

It has been previously mentioned in other research reports that neck circumference represents certain advantages over other indicators, such as BMI or waist circumference [13,28,35]. Neck circumference is an adiposity indicator for the upper body segment, and it is not necessarily repeated to obtain a reliable measurement, as it can be taken at any time of the day without variation.

One limitation of this study might be the fact that the sample size was selected by convenience. Furthermore, it is important to point out that although this study included a considerable sample size, due to the differences in body composition related to the diversity of the Mexican population, the data do not represent the entire country’s population. Notably, although we adopted the WHO recommendations to create a pattern reference, we included only the BMI/age standard to classify normal weight children. 

## 5. Conclusions

This study presents the first reference percentile values for the neck circumference of Mexican children, which may be applied to the identification of subjects far from the mean as well as for clinical follow-up.

## Figures and Tables

**Table 1:** Anthropometric measurements of schoolchildren aged 6–11 years in Acatlán de Juárez and Villa Corona Jalisco, México.

Gender	Age (years)	*n*	Weight (kg)		Height (cm)		BMI (kg/m^2^)		WC (cm)		NC (cm)		BF (%)	
			Mean	SD	Mean	SD	Mean	SD	Mean	SD	Mean	SD	Mean	SD
Female	6	114	20.67	2.81	116.33	5.52	15.16	1.19	52.24 +	3.42	24.61 *	1.08	15.42 *	4.12
	7	99	23.60	3.89	123.16	5.50	15.33	1.16	54.01	3.11	25.25 *	1.14	15.77 *	3.58
	8	94	25.72	3.60	127.75 +	5.51	15.71	1.45	55.54	3.58	25.79 *	1.32	16.91 *	4.50
	9	92	29.54	3.81	134.39	6.40	16.31	1.33	58.00	3.43	26.50 *	1.18	18.43 *	3.91
	10	90	33.04	4.90	141.00	7.32	16.51	1.45	59.66	4.52	27.07 *	1.19	18.62 +	4.15
	11	71	39.42 +	6.95	148.90 +	7.58	17.67	2.06	62.99	5.11	28.40 +	1.59	20.70 *	4.47
Male	6	91	21.12	2.47	117.30	5.14	15.21	0.93	53.32 +	2.69	25.74 *	1.10	13.18 *	2.84
	7	85	23.54	2.62	123.09	5.03	15.51	1.08	54.64	3.12	26.28 *	1.26	13.68 *	3.65
	8	76	26.60	3.06	129.66 +	5.29	15.78	1.08	56.48	3.25	27.06 *	1.05	14.07 *	3.22
	9	92	29.44	3.87	134.64	5.73	16.18	1.38	58.30	4.26	27.40 *	1.28	14.47 *	4.23
	10	85	33.12	5.22	140.33	5.63	16.74	1.91	60.93	5.46	28.10 *	1.50	16.59 +	5.42
	11	70	36.74 +	5.44	145.77 +	6.30	17.18	1.71	62.65	4.69	28.96 +	1.39	17.46 *	5.75

BMI: body mass index; WC: waist circumference; NC: neck circumference; BF: body fat; SD: standard deviation. * represents the statistical differences (*p* < 0.001) for one anthropometric parameter between males and females of the same age. + represents the statistical differences (*p* < 0.05) for one anthropometric parameter between males and females of the same age.

**Table 2:** Correlation coefficients between neck circumference and adiposity anthropometric indicators by sex and age.

Sex	Age	*n*	BMI	WC	BF (%)	TSF	SSF	*p*
Female	6	114	0.51	0.60	0.35	0.37	0.34	<0.001
	7	99	0.62	0.65	0.43	0.45	0.30	<0.001
	8	94	0.63	0.62	0.49	0.49	0.44	<0.001
	9	92	0.53	0.59	0.32	0.34	0.24	<0.001
	10	90	0.63	0.65	0.36	0.31	0.32	<0.001
	11	71	0.65	0.70	0.52	0.42	0.55	<0.001
Male	6	91	0.59	0.67	0.53	0.48	0.47	<0.001
	7	85	0.53	0.54	0.42	0.38	0.41	<0.001
	8	76	0.56	0.60	0.33	0.29	0.33	<0.001
	9	92	0.67	0.70	0.57	0.49	0.57	<0.001
	10	85	0.84	0.84	0.73	0.68	0.77	<0.001
	11	70	0.65	0.72	0.24	0.26	0.22	<0.001

BMI: body mass index; WC: waist circumference; BF: body fat; TSF: tricipital skinfold thickness; SSF: subscapular skinfold thickness.

**Table 3:** Percentile distribution of neck circumference (cm) of schoolchildren aged 6–11 years in Acatlán de Juarez and Villa Corona Jalisco, México.

Percentiles													
Age (years)	L	M	S	3	5	10	25	50	75	85	90	95	97
Female													
6	0.1292	24.6009	0.0435	22.66	22.89	23.26	23.89	24.60	25.33	25.73	26.01	26.42	26.69
7	−1.4136	25.1297	0.0447	23.21	23.43	23.78	24.40	25.13	25.92	26.36	26.68	27.16	27.48
8	−2.4229	25.7153	0.0460	23.77	23.99	24.34	24.96	25.72	26.56	27.05	27.40	27.96	28.34
9	−2.0373	26.3952	0.0474	24.32	24.55	24.93	25.59	26.40	27.28	27.80	28.16	28.73	29.12
10	−0.5193	27.2256	0.0490	24.88	25.16	25.59	26.35	27.23	28.15	28.66	29.02	29.56	29.92
11	1.0549	28.2507	0.0507	25.55	25.89	26.41	27.28	28.25	29.22	29.73	30.08	30.60	30.94
Male													
6	−0.1859	25.7458	0.0431	23.76	23.99	24.37	25.01	25.75	26.51	26.93	27.22	27.65	27.94
7	−0.7149	26.2513	0.0439	24.23	24.47	24.84	25.49	26.25	27.05	27.49	27.80	28.27	28.58
8	−1.2831	26.8052	0.0449	24.74	24.98	25.36	26.02	26.81	27.65	28.12	28.46	28.97	29.31
9	−1.8881	27.4073	0.0459	25.30	25.54	25.92	26.60	27.41	28.30	28.81	29.17	29.73	30.12
10	−2.5277	28.0587	0.0470	25.91	26.15	26.53	27.22	28.06	29.00	29.56	29.95	30.58	31.01
11	−3.2002	28.7605	0.0482	26.56	26.80	27.18	27.89	28.76	29.76	30.37	30.81	31.51	32.01

L: lambda, M: mean, S: variation coefficient.

## Data Availability

Data supporting reported results are available upon request to the corresponding author.
